# Efficacy of histamine H1 receptor antagonists azelastine and fexofenadine against cutaneous *Leishmania major* infection

**DOI:** 10.1371/journal.pntd.0008482

**Published:** 2020-08-10

**Authors:** Alex G. Peniche, E. Yaneth Osorio, Peter C. Melby, Bruno L. Travi

**Affiliations:** 1 Department of Internal Medicine, University of Texas Medical Branch, Galveston, Texas, United States of America; 2 Center for Tropical Diseases, University of Texas Medical Branch, Galveston, Texas, United States of America; Pasteur Institute of Iran, ISLAMIC REPUBLIC OF IRAN

## Abstract

Current drug therapies for cutaneous leishmaniasis are often difficult to administer and treatment failure is an increasingly common occurrence. The efficacy of anti-leishmanial therapy relies on a combination of anti-parasite activity of drugs and the patient’s immune response. Previous studies have reported *in vitro* antimicrobial activity of histamine 1-receptor antagonists (H1RAs) against different pathogens. We used an *ex vivo* explant culture of lymph nodes from mice infected with *Leishmania major* to screen H1RAs compounds. Azelastine (AZ) and Fexofenadine (FX) showed remarkable *ex vivo* efficacy (EC_50_ = 0.05 and 1.50 μM respectively) and low *in vitro* cytotoxicity yielding a high therapeutic index. AZ significantly decreased the expression of H1R and the proinflammatory cytokine *IL-1ẞ* in the *ex vivo* system, which were shown to be augmented by histamine addition. The anti-leishmanial efficacy of AZ was enhanced in the presence of T cells from infected mice suggesting an immune-modulatory mechanism of parasite suppression. *L*. *major* infected BALB/c mice treated per os with FX or intralesionally with AZ showed a significant reduction of lesion size (FX = 69%; AZ = 52%). Furthermore, there was significant parasite suppression in the lesion (FX = 82%; AZ = 87%) and lymph nodes (FX = 81%; AZ = 36%) with no observable side effects. AZ and FX and potentially other H1RAs are good candidates for assessing efficacy in larger studies as monotherapies or in combination with current anti-leishmanial drugs to treat cutaneous leishmaniasis.

## Author summary

Cutaneous leishmaniasis (CL) is a parasitic disease present in more than 90 countries. Different species of *Leishmania* produce skin ulcers upon infection through the bite of infected sand fly vectors. There are several drugs used to treat CL but most of them are toxic or difficult to administer and there is increasing drug resistance leading to treatment failure. Therefore, new drugs are needed for treating CL. The objective of this study was to determine the anti-leishmanial efficacy of antihistamine drugs. Using cell cultures of lymph nodes obtained from *Leishmania major* infected mice, we evaluated the parasiticidal activity of the antihistamine drugs azelastine and fexofenadine. Both drugs showed high efficacy against *L*. *major* and low toxicity for a human cell line. Treatment of mice infected in the skin with *L. major* indicated that both azelastine and fexofenadine significantly reduced the size of the lesions and suppressed parasite multiplication. Consequently, these two drugs are good candidates to further evaluate their efficacy as monotherapies or in combination with other anti-leishmanial drugs.

## Introduction

The leishmaniases are a group of diseases reported in >95 countries across five continents [1]. The disease is caused by the protozoan *Leishmania* and is transmitted to humans by the bite of different species of phlebotomine sand flies [2]. Cutaneous leishmaniasis is endemic in many countries of the Old and New World affecting between 600,000 and 1 million people worldwide (https://www.who.int/leishmaniasis/en/). Current systemic treatments (sodium stibogluconate, pentamidine, miltefosine, amphotericin B) are difficult to administer, have high toxicity and the frequent appearance of drug-resistant parasites have resulted in increasing numbers of unresponsive individuals [3–9]. In addition to drug-resistant *Leishmania*, there is evidence that the immune response plays an important role in the outcome of treatment [10–12]. Consequently, the identification of new, less toxic anti-leishmanial drugs that have a direct effect on *Leishmania* or promote efficient parasite killing through immunological enhancement is an urgent need.

The repurposing of FDA-approved drugs is a faster and more cost-effective approach to identify new therapies compared with conventional screening of new molecules using methods based on molecular targets or phenotypic evaluations. It is estimated that FDA approval for repurposed drugs could take 2–3 years and around 10 million USD, while the conventional approaches require approximately 1 billion USD and 10 to 12 years [13].

Among potential anti-leishmanial candidates, anti-histamine drugs commonly used to treat allergies could be thoroughly evaluated due to their reported activity against a wide variety of pathogens. Histamine 1-receptor antagonists (H1RAs) have shown activity against *Mycobacterium tuberculosis* [14], *Plasmodium falciparum* [15–17] and *Litomosoides sigmodontis* [18]. In addition, there is indication that second generation H1RAs have *in vitro* and *in vivo* activity against *Leishmania infantum* [19, 20]. Therefore, H1RAs are an attractive group of compounds for evaluation of treatment of cutaneous leishmaniasis, either alone or in combination with existing anti-leishmanial drugs. An understanding of their mode of action should complement the empirical evaluation of their anti-leishmanial efficacy. This information will contribute to optimization of therapeutic efficacy of the most active H1RAs and the design of additional analogues with maximum potency.

This study evaluated the anti-leishmanial activity of a small collection of FDA-approved H1RAs from diverse generations and chemotypes with the purpose of identifying new molecules to treat cutaneous leishmaniasis. The evaluations were performed using the *ex vivo* lymph node explant model that we have previously developed for *L*. *major* [21]. In this system, the infected cells are obtained from the draining lymph nodes of *L*. *major*-infected mice. Thus, the cell culture where the drug has to exert its activity mimics the environment of *Leishmania* infection, which contains amastigote-laden macrophages, dendritic cells, lymphocytes and secreted cytokines. The *ex vivo* and *in vivo* results from BALB/c mice infected with *L*. *major* indicated that azelastine (AZ) and fexofenadine (FX) have significant anti-leishmanial efficacy that warrants further studies with these anti-histamine compounds.

## Materials and methods

### Ethics statement

The procedures involving animals were approved by the Institutional Animal Care and Use Committee (IACUC protocol: 1011058) of the University of Texas Medical Branch. UTMB IACUC adheres to the Public Health Service Policy on Humane Care and Use of Laboratory Animals 2002, reprint 2015. U.S. government Principles for the Utilization and Care of Vertebrate Animals Used in Testing, Research, and Training Guide for the Care and Use of Laboratory Animals, 8th Edition; AVMA Guidelines for the euthanasia of Animals 2013.

### Animals and parasites

The procedures involving animals were approved by the Institutional Animal Care and Use Committee (IACUC protocol: 1011058) of the University of Texas Medical Branch. UTMB IACUC adheres to the Public Health Service Policy on Humane Care and Use of Laboratory Animals 2002, reprint 2015. U.S. government Principles for the Utilization and Care of Vertebrate Animals Used in Testing, Research, and Training Guide for the Care and Use of Laboratory Animals, 8th Edition; AVMA Guidelines for the euthanasia of Animals 2013.

Eight-week old male BALB/c (genotype AnNHsd) mice were purchased from Harlan Laboratories (Indianapolis, IN) and used in all the experiments. *L*. *major* (MHOM/IL/81/Friedlin) promastigotes were transfected with an episomal vector containing the luciferase (LUC) reporter gene [21]. Promastigotes were cultured at 28°C in M199 (Gibco, Grand Island, NY) supplemented with 0.12 mM adenine, 0.0005% hemin, and 20% fetal bovine serum [FBS]). Geneticin (10 μg/mL, Gibco) was added to the culture medium to select for LUC-carrying promastigotes. *L*. *major* virulence was maintained by passage through mice every 2 to 3 months, and parasites recovered from these animals were used for *in vitro* determinations.

### Histamine 1-receptor antagonist compounds

We tested a collection of 11 H1RAs identified by Compound Identification Number (CID) in Pubchem (**S1 Fig**). All compounds were obtained from Sigma (St. Louis, MO), except for AZ which was acquired from AK Scientific (Union City, CA). All compounds were dissolved in cell culture-tested dimethyl sulfoxide (DMSO) (Sigma) at a stock concentration of 20 mM and stored in aliquots at -20°C. Miltefosine and amphotericin B (Sigma) were used as positive controls of anti-leishmanial activity. Vehicle controls with equivalent DMSO concentrations were used as untreated reference in all experiments.

### Anti-leishmanial *in vitro* efficacy of H1R antagonists

An *ex vivo* explant culture (EVC) used in multiple experiments was obtained as previously described [21]. Briefly, the mice were inoculated on the snout and rump with 10^7^ metacyclic promastigotes [22] of *L*. *major-*LUC. At 3 weeks post-infection (p.i.), the draining lymph nodes (retropharyngeal and sub-iliac) of infected animals were aseptically removed, infiltrated with 2 mg/mL of collagenase D (Roche) and incubated for 30 minutes at 37°C [21]. The cell suspension was washed in DMEM, and re-suspended in 2X supplemented culture medium, composed of DMEM (Cellgro), 10% heat-inactivated FBS (Atlanta Biologicals, Lawrenceville, GA), 2 mM Sodium pyruvate (Sigma, St. Louis MO), 2X MEM amino acids solution (Sigma), and 20 mM HEPES buffer (Cellgro). The EVC 100,000-cell suspension in 100 μL was dispensed in white luminometry plates (Costar) and exposed to 2-fold serial dilutions of 2X H1RAs in 100 μL DMEM (plain culture medium).

To calculate the concentration of compound that killed 50% of the parasites (EC_50_), we determined the parasite burden by luminometry after 48 h of culture at 34°C [21]. Cells exposed to compounds were lysed and the luciferase signal read in a plate luminometer (FLUOstar Omega, BMG Labtech) after adding 100 μL of luciferin substrate (Promega). The percentage of parasite inhibition compared with the vehicle control was calculated as = 100 - [(parasite counts in treated cells/parasite counts in vehicle wells) x 100]. The EC_50_ was determined by regression analysis using GraphPad (Prism 5.0) and the mean and standard error from three different experiments was utilized to estimate the final EC_50_. The anti-leishmanial efficacy of compounds was evaluated using *L*. *major*-LUC promastigotes in the logarithmic phase of growth (4 days). The parasites were grown in M199 supplemented with 20% FBS, 0.1% hemin and 10 mM adenine. Then, *L*. *major* was harvested and exposed to serial 2-fold dilutions of the compounds in a 100μL volume (10^6^ promastigotes/mL) in 96-well white plates. After 48 h at 28°C incubation, the parasites were pelleted by centrifugation, lysed and 100 μL of luciferin substrate was added (Promega). The luciferase signal was read as described above.

The anti-leishmanial efficacy of AZ was also evaluated in adherent peritoneal macrophages from naïve mice infected *in vitro* with *L*. *major* promastigotes (1 cell: 10 parasites) during 4 h at 34°C. After washing extracellular parasites, infected cells were transferred to white plates (10,000/well) and exposed to AZ. The effect of T cells on parasite burden of AZ-treated cells was evaluated using CD3T+ cells. These cells were isolated by positive selection with magnetic beads (MojoSort, Biolegend) from the lymph nodes of either uninfected or *L*. *major*-infected mice (1 infected macrophage: 2 lymphocytes ratio). Parasite burden was determined after 48 h of culture by luminometry.

### Determination of cytotoxicity and calculation of *in vitro* therapeutic index (IVTI)

To determine compound toxicity we used the HepG2 cell line (human hepatocellular carcinoma, ATCC HB-8065) as a widely used cell-based assay [23, 24]. The cells were maintained in MEM (Gibco) supplemented with 5% heat-inactivated FBS, 1 mM Sodium pyruvate (Gibco) and 1X MEM amino acids solution (Sigma). Briefly, cells were added to white-bottomed 96-well plates containing 100 μL of serial 2-fold dilutions of the H1RAs. After 24 hours of culture at 37ºC, the percentage of viable cells was determined by ATP quantification using the CellTiter-Glo luminescent Cell Viability Assay (Promega) according to the manufacturer’s instructions. The percentage of cytotoxicity compared with controls allowed the use of a regression model to calculate the cytotoxic concentration that killed 50% of the cells (CC_50_) using GraphPad (Prism 5.0). At least three different assays were carried out to determine the IVTI of each compound, which was calculated as the ratio between the CC_50_ obtained in the HepG2 cell line and the EC_50_ determined in the *L*. *major* EVC [21]. To evaluate the cytotoxicity of AZ and FX in peritoneal macrophages, adherent cells obtained from peritoneal lavage of naïve mice (10,000/well) were exposed during 24 hours at 37°C to serial drug concentrations (0.09 μM—200 μM). Cell viability was determined by CellTiter-Glo (Promega).

### Gene expression by qPCR

After 48 h of *ex vivo* treatments, the samples were lysed in 10 μL of IGEPAL lysis buffer (10 mM Tris-HCL ph7.4 + 0.3% IGEPAL, 0.1% BSA + 150 mM NaCl and 1,500 cells/μL) [25] and reverse transcribed (High-Capacity cDNA Reverse Transcription Kit, ThermoFisher Sci.). Mouse *h1r*, *il-1ẞ*, and *Leishmania 18s* genes were amplified by qPCR using the following primers: mouse *h1r*: Fw- CAAGATGTGTGAGGGGAACAG; Rev-CTACCGACAGGCTGACAATGT (PrimerBank database; ID and 31542963a1) [26]; Mouse *il-1β*: Fw- TTGACGGACCCCAAAAGATG; Rev- AGAAGGTGC TCATGTCCTCAT) [27]; *Leishmania 18s*: Fw-CCAAAGTGTGGAGATCGAAG; Rev- GGCCGGTAAAGGCCGAATAG [28]. Amplicons were detected with SYBR. The fold change of gene expression was estimated by the delta CT method using the host cell *18s* as reference gene. The percentage of parasite load was calculated with reference to DMSO controls.

### Evaluation of *in vivo* efficacy

We used groups of 7 mice in our experiments because this group size had 80% power (alpha 0.05 significance) to detect 90% reduction in parasite load in treated groups versus the untreated control group (vehicle only). Mice were infected in the rump with 10^7^ opsonized promastigotes of *L*. *major*-LUC as we previously described [21]. The animals were treated starting at day 3 p.i. for up to 10 days. AZ (0.0625 and 0.125 mg/mouse) and FX (40 and 80 mg/kg) were dissolved in sterile PBS and administered by intralesional injection (ID) or gavage (PO), respectively. Mice receiving AZ were treated 3 times at 48-hour intervals. Control mice received sterile PBS according to the route used by each H1RAs (ID or PO). Miltefosine was administered orally at doses of 25 mg/kg or 50 mg/kg for 10 days and used as positive control of parasite suppression. The clinical efficacy was assessed by comparing lesion size (LS) after 10 days of treatment compared with the negative vehicle control as described by Grogl et al. [29]. Therefore, percent suppression was defined as [(LS negative control–LS compound) / LS negative control] x 100. A parasite suppression ≥50% compared with the untreated control was considered significant anti-leishmanial activity. Lesion size (area in mm^2^ = length x width) was measured using a digital caliper (Mitutoyo; Kawasaki, Japan).

The parasite burden of individual mice was assessed *in vivo* at the beginning and end of the experiment using the IVIS Spectrum equipment (Perkin Elmer) by injecting the animals with 1.5 mg luciferin (GoldBio) intradermally at the infection site. The ultrasensitive IVIS camera quantitatively measures the light emitted by the luciferase-transfected *L*. *major* upon exposure to the substrate (firefly luciferin). The mice were imaged under deep anesthesia using an isoflurane vaporizer. They were placed in a supine position in the pre-warmed IVIS box and the images were taken exactly 5 minutes after luciferin injection. The analysis was performed after defining a standard region of interest (ROI) over the infection site using the Living Image software (Xenogen Corporation, Almeda, CA). This protocol allowed us to determine the parasite burden in the same animal over multiple time points. Results were expressed in units of photons per second. A compound was considered effective *in vivo* when it reduced ≥80% the parasite luminescent signal compared to the control untreated group. This approach is in accordance with the criteria proposed for *Leishmania* in deciding when a compound qualifies to progress into ‘lead’ compound identification [30].

### Statistical analysis

Data were analyzed using InStat (v. 3.0) and Prism 5.0 (Graphpad, La Jolla CA). The *in vitro* efficacy and cytotoxicity was determined by regression analysis using the least squares method. Tests were chosen according to data distribution following software recommendations. Statistical tests and number of animals are described in figure legends.

## Results

### Efficacy and toxicity of H1R antagonists using the *L*. *major*-ex vivo lymph node explant

**Fig 1:**
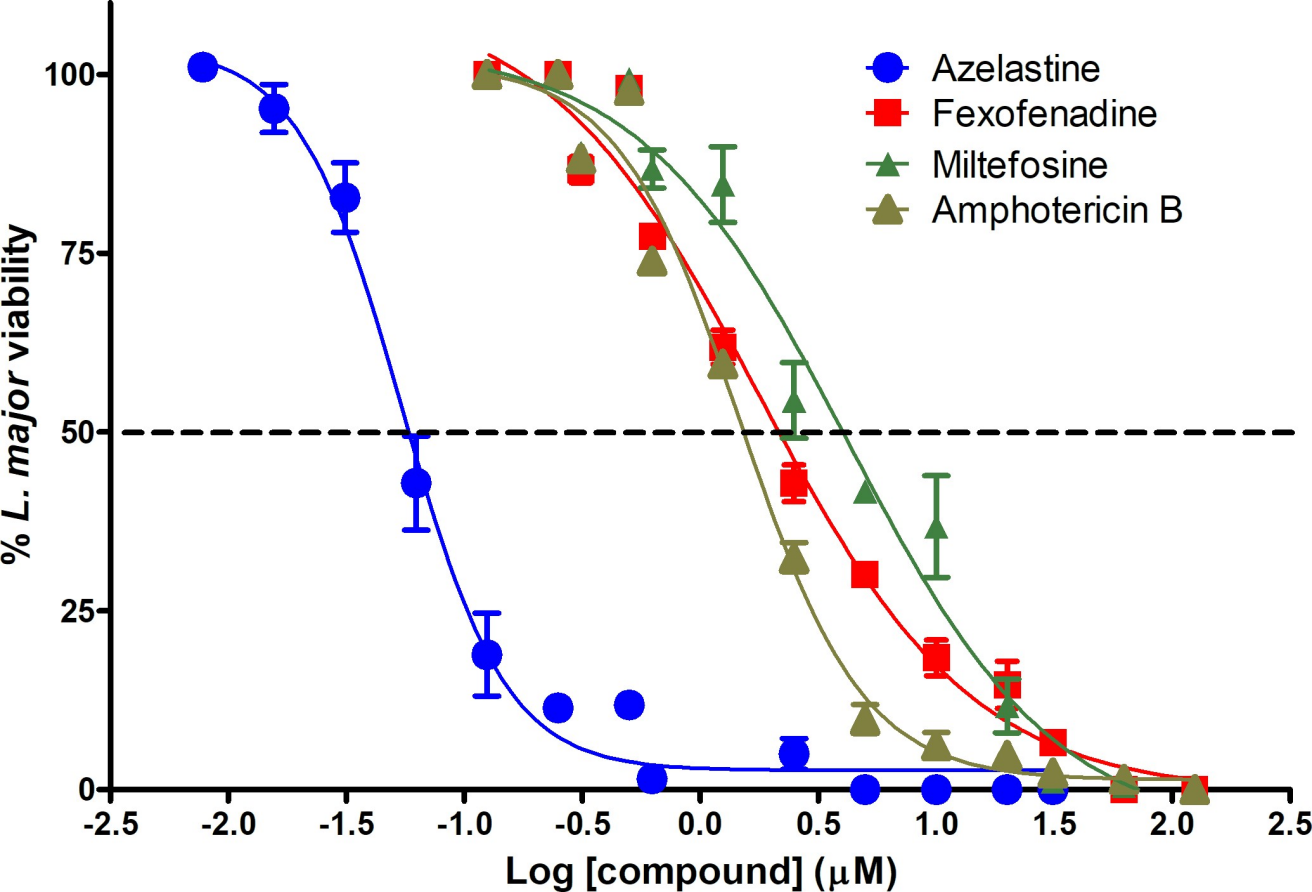
Suppression of *L*. *major* upon exposure to azelastine, fexofenadine and the anti-leishmanial drugs amphotericin B and miltefosine using the ex vivo system.

**Fig 2:**
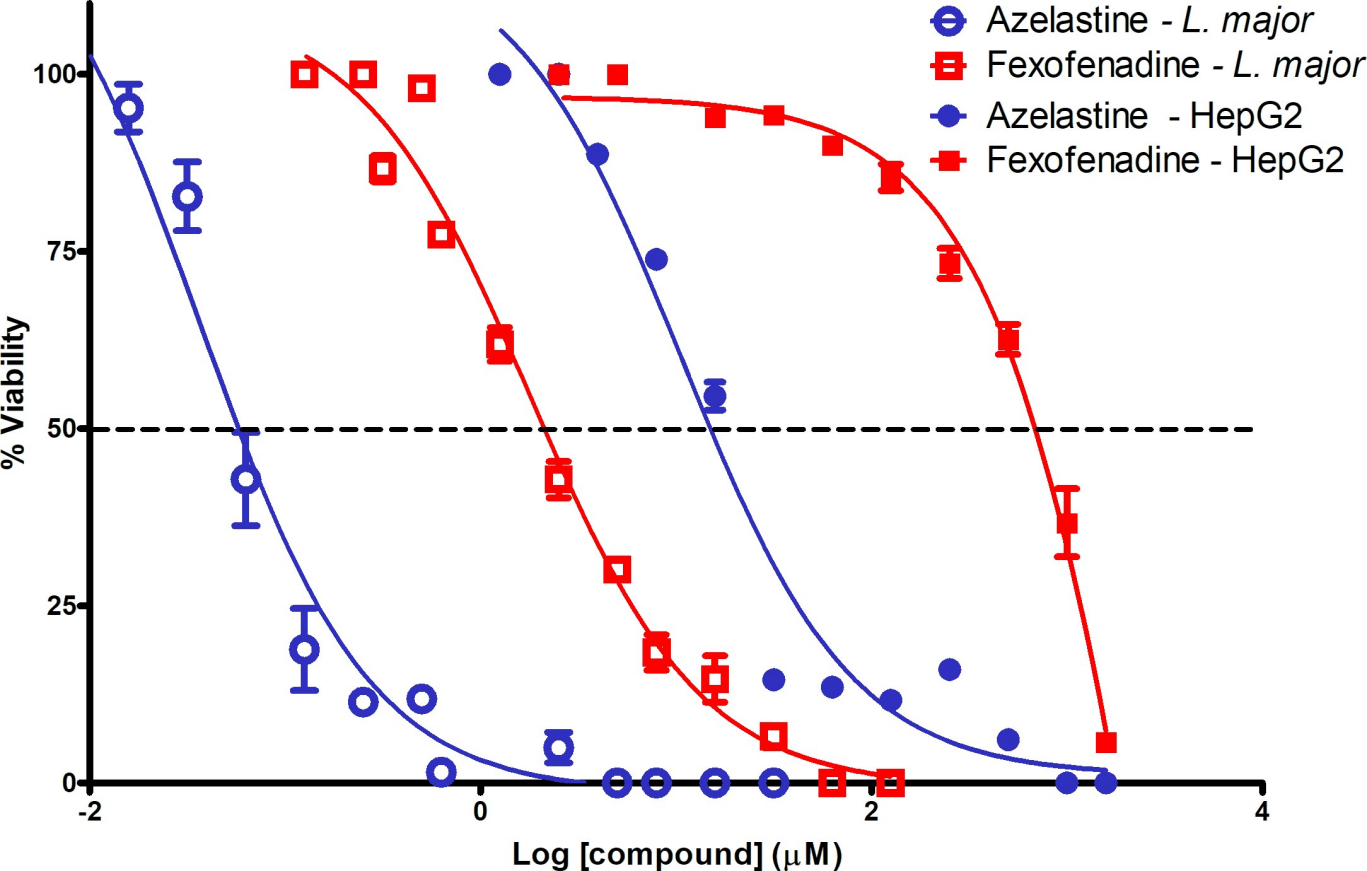
Anti-leishmanial efficacy (EC_50_) versus cytotoxicity (CC_50_) for determining the *in vitro* therapeutic index of azelastine and fexofenadine. The EC_50_ was determined in the *ex vivo* system while the CC_50_ was assessed using HepG2 cells.

The evaluation of 11 H1RAs using the *L*. *major* EVC showed that AZ was the most active compound (EC_50_ = 0.05 ± 0.02 μM). FX, also demonstrated good anti-leishmanial activity (EC_50_ = 1.50 ± 0.18 μM) in the EVC. The efficacy of these compounds was achieved at concentrations similar or lower than known anti-leishmanial compounds (miltefosine and amphotericin B) (**Fig 1**). In addition, chloropyramine, cyproheptadine and mequitazine had appreciable *in vitro* potency with an EC_50_ <10 μM (range = 3.87 to 7.44 μM) (**Table 1**). The cytotoxic concentration (CC_50_) of compounds was determined in the mammalian cell line HepG2 (**Table 1**). We ranked the therapeutic potential of compounds using the results obtained after calculating the *in vitro* therapeutic-index (IVTI). Only AZ and FX showed excellent therapeutic potential against *L*. *major* according to their remarkably high IVTI values (**Table 1 and Fig 2**). Chloropyramine, Cyproheptadine and Mequitazine ranked lower because, despite having an acceptable EC_50_, their cell toxicity rendered lower IVTIs (range 11–36) (**Table 1**). We also evaluated AZ and FX CC_50_ using peritoneal macrophages. AZ and FX toxicity in the highly sensitive mouse peritoneal macrophages (compared with HepG2 standard cells) was 0.95 μM and ≥100 μM, respectively. These results corresponded to IVTIs of 19 and ≥66, respectively.

**Table 1:** *In vitro* therapeutic index of chemically diverse histamine H1 receptor antagonists (H1RAs) determined in the *ex vivo* lymph node explant system of *L*. *major* for anti-leishmanial activity and HepG2 cells for cytotoxicity.

Compound	CID^1^	EC_50_^2^ (μM)	CC_50_^3^ (μM)	IVTI	Chemotype Class	Reported antimicrobial activity^5^
Azelastine	2267	0.05 ± 0.02	45.1 ± 11.20	942	Phthalazine	*T*. *cruzi*
Fexofenadine	63002	1.50 ± 0.18	822.2 ± 87.20	541	Piperidine	None
Chloropyramine	80311	3.87 ± 0.20	140.3 ± 49.60	36	Ethylenediamine	*L*. *major*
Triprolidine	5702129	11.23 ± 4.20	389.5 ± 27.90	35	Pyridine	*P*. *falciparum*
Ketotifen	3827	11.86 ± 0.10	282.4 ± 22.90	24	Thiophene	*P*. *falciparum*
Cyproheptadine	2913	7.44 ± 2.60	171.7 ± 41.00	23	Dibenzocycloheptene	*M*. *tuberculosis*, *P*. *yoelii*
Diphenhydramine	3100	19.73 ± 4.40	375.2 ± 17.80	19	Benzhydryl	*P*. *falciparum*
Mequitazine	4066	4.53 ± 3.90	50.3 ± 8.75	11	Phenothiazine	*P*. *falciparum*, *T*. *cruzi*, *T*. *brucei*, *S*. *Typhimurium*
Dexchlorpheniramine maleate	5281070	35.18 ± 6.90	176.9 ±16.30	5	Chlorpheni-ramine	*P*. *falciparum*
Mianserin	4184	37.04 ± 4.20	156.8 ± 19.00	4	Dibenzazepine	None
Methapyrilene	8667	>100	579.2 ± 20.60	NC	Ethylenediamine	*P*. *falciparum*
Miltefosine		1.64 ± 0.26	74.94 ± 5.01	43	-	Positive Control: current ntileishmanial drug
Amphotericin B		0.50 ± 0.01	16.27 ± 1.49	33	-	Positive Control: current anti-leishmanial drug

^1^CID: Compound identifier (PubChem)

^2^The data represent means from two to three different experiments performed using luminescence to calculate the 50% effective concentration (EC_50_) in *L*. *major ex vivo* system.

^3^The 50% cytotoxicity (CC_50_) was determined using the HepG2 cell line

^4^The IVTI was calculated as the ratio between the CC_50_ and EC_50_ of each compound. The EC_50_ was determined by regression analysis using GraphPad (Prism 5.0) software.

^5^Actimicrobial activity as reported by Pubchem-Bioassays

**Fig 3:**
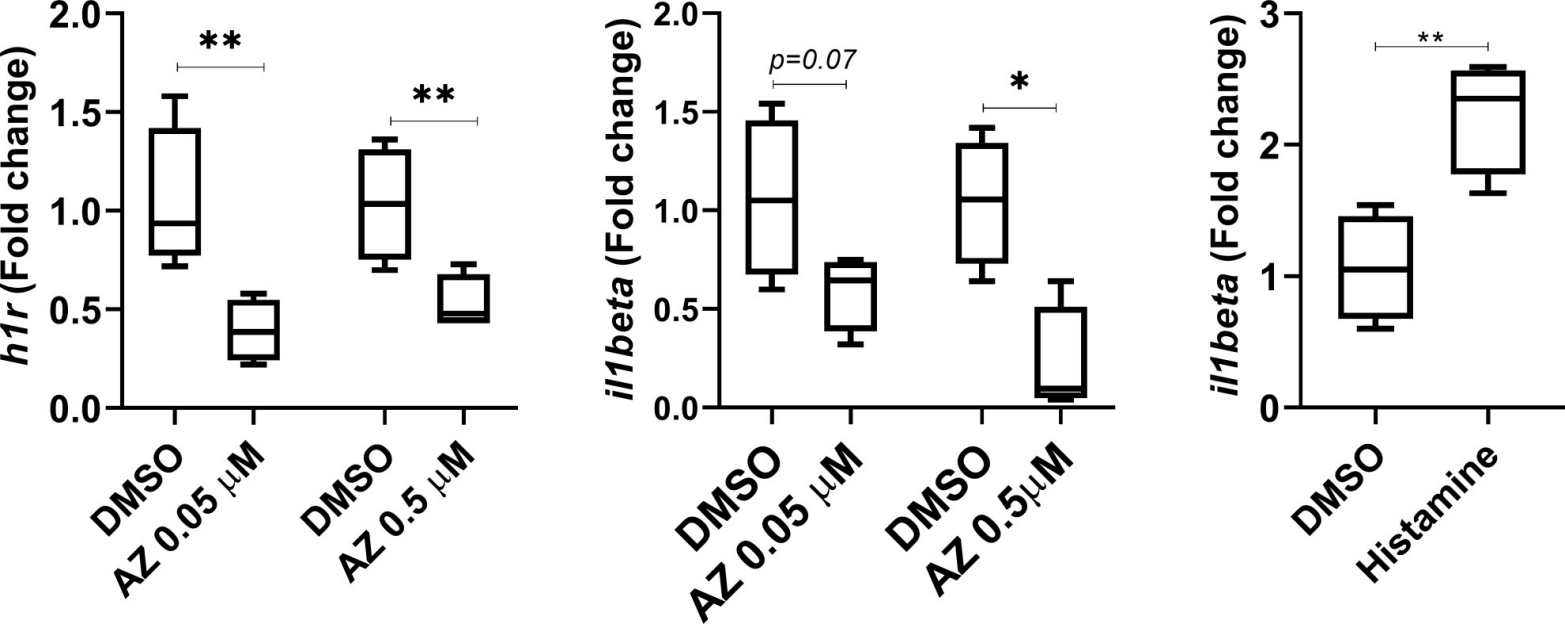
Decrease of histamine receptor 1 (*HR1*) and proinflammatory *IL-1ẞ* expression by addition of azelastine to an *ex vivo* lymph node explant culture of BALB/c mice infected with *Leishmania major*. Addition of histamine increased the expression of *IL-1ẞ*. Determinations were made using quantitative PCR. (*p = 0.05; **p = 0.01).

**Fig 4:**
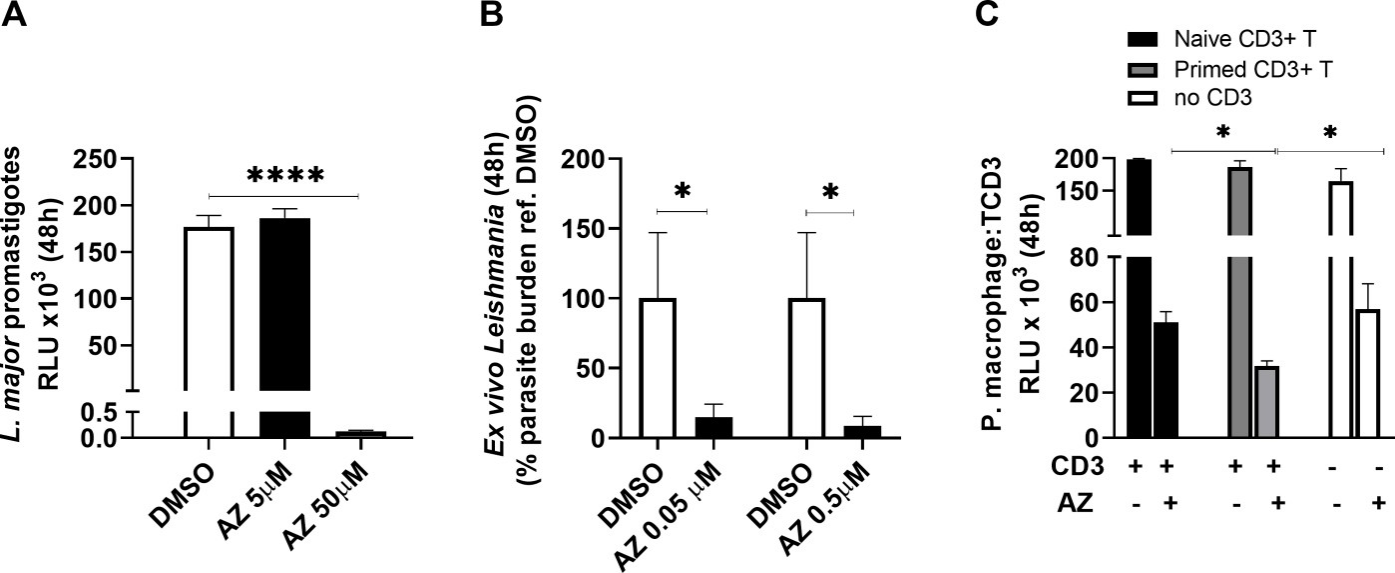
Activity of azelastine (AZ) against *L*. *major*. (A) promastigote numbers of *L*. *major*-LUC after 48 h of incubation at 26°C exposed to either AZ or DMSO quantified by luminometry as relative light units (RLU; ****p<0.0001, Tukey’s multiple comparison test). (B), quantification of *L*. *major* in *ex vivo* cultures from infected mice using *Leishmania 18s* gene expression after 48 h of AZ exposure at 34°C (data expressed with reference to untreated DMSO controls;. *p = 0.02, Mann-Whitney test). (C), anti-leishmanial activity of AZ in *L*. *major*-infected mouse peritoneal macrophages co-cultured with either naïve lymphocytes (black bars), *Leishmania*-primed lymphocytes (gray bars) or without lymphocytes (empty bars). Parasite burden was determined by luminometry (RLU) after 48 h of incubation at 34°C (*p<0.05, Tukey’s multiple comparisons test).

Additional assays using the EVC showed that histamine markedly increased the expression of H1R while AZ significantly inhibited its expression and reduced the pro-inflammatory cytokine *IL1-ẞ* as determined by quantitative PCR **(Fig 3).** We also found a tendency of AZ to decrease the expression of IL-6 (20.1± 21%) but this was highly variable. AZ had suppressive effect against cultured promastigotes only when used at concentrations 100-fold higher than those used against intracellular amastigotes (**Fig 4**). Naïve mouse peritoneal macrophages infected *in vitro* with *L*. *major* and co-cultured with purified T cells from infected or uninfected mice suggested that AZ at 0.5 μM concentration had a positive effect on intracellular amastigote suppression. Furthermore, the addition of primed T cells from infected mice significantly enhanced parasite killing compared with macrophages alone or co-cultured with naïve lymphocytes in the presence of AZ **(Fig 4).**

### *In vivo* efficacy of H1RA compounds in the mouse model

**Fig 5:**
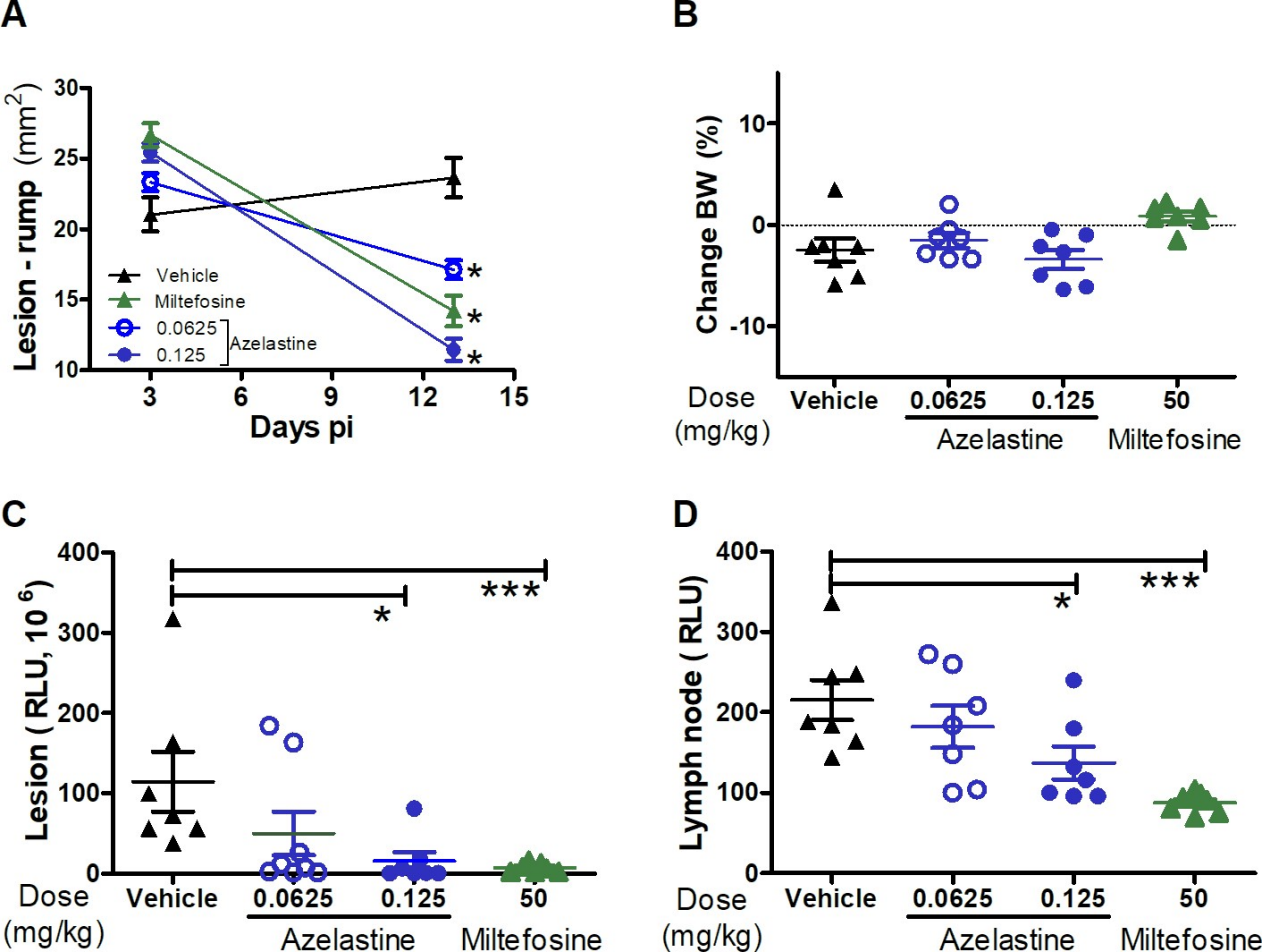
Evaluation of azelastine efficacy in BALB/c mice infected with *L*. *major*. Mice (n  =  7) were infected intradermically (ID) with 10^7^ metacyclic promastigotes of *L*. *major* transfected with the luciferase gene. Lesion size (area in mm2 = length x width) was measured using a digital caliper (A). Body weight change was estimated as a major sign of toxicity (B). The parasite load at the infection site (C) and draining lymph nodes (D) was determined *in vivo* using the IVIS spectrum imager. Animals received 3 ID injections of azelastine or vehicle (control) from day 3 to 10 p.i. Another group of mice was treated orally with miltefosine 50 mg/kg for 10 days as a positive control of parasite suppression. The figures show mean values and their standard deviation (SD). Representative data of 2 independent experiments. P value: *< 0.05; ***< 0.001. The statistical significance of the data was determined using the t test.

We selected AZ and FX for preclinical because they showed the highest IVTI in the EVC assays. The *in vivo* readouts of drug efficacy were the reduction of parasite load at the infection site and draining lymph nodes and the decrease of the lesion size compared with the untreated controls. Mice were treated between days 3 and 13 p.i., which is considered the early phase of infection, when an adaptive immune response is being established [31, 32]. The mice treated every other day with three intralesional injections of AZ (0.125 mg/mouse) at the infection site showed a 52% lesion diminution compared with untreated controls (**Fig 5A**). None of the mice treated with this compound presented weight loss or any observable side effect during treatment (**Fig 5B**). This treatment protocol significantly reduced the parasite load in the lesion (87%, p = 0.024) and lymph node (36%, p = 0.031) compared to untreated controls as determined by luminometry (**Fig 5C and 5D**). The lower dose of AZ (0.06 mg/mouse) produced a statistically non-significant reduction of the parasite burden in the lesion (57%) and lymph node (15%) compared with the control group (**Fig 5C and 5D**).

**Fig 6:**
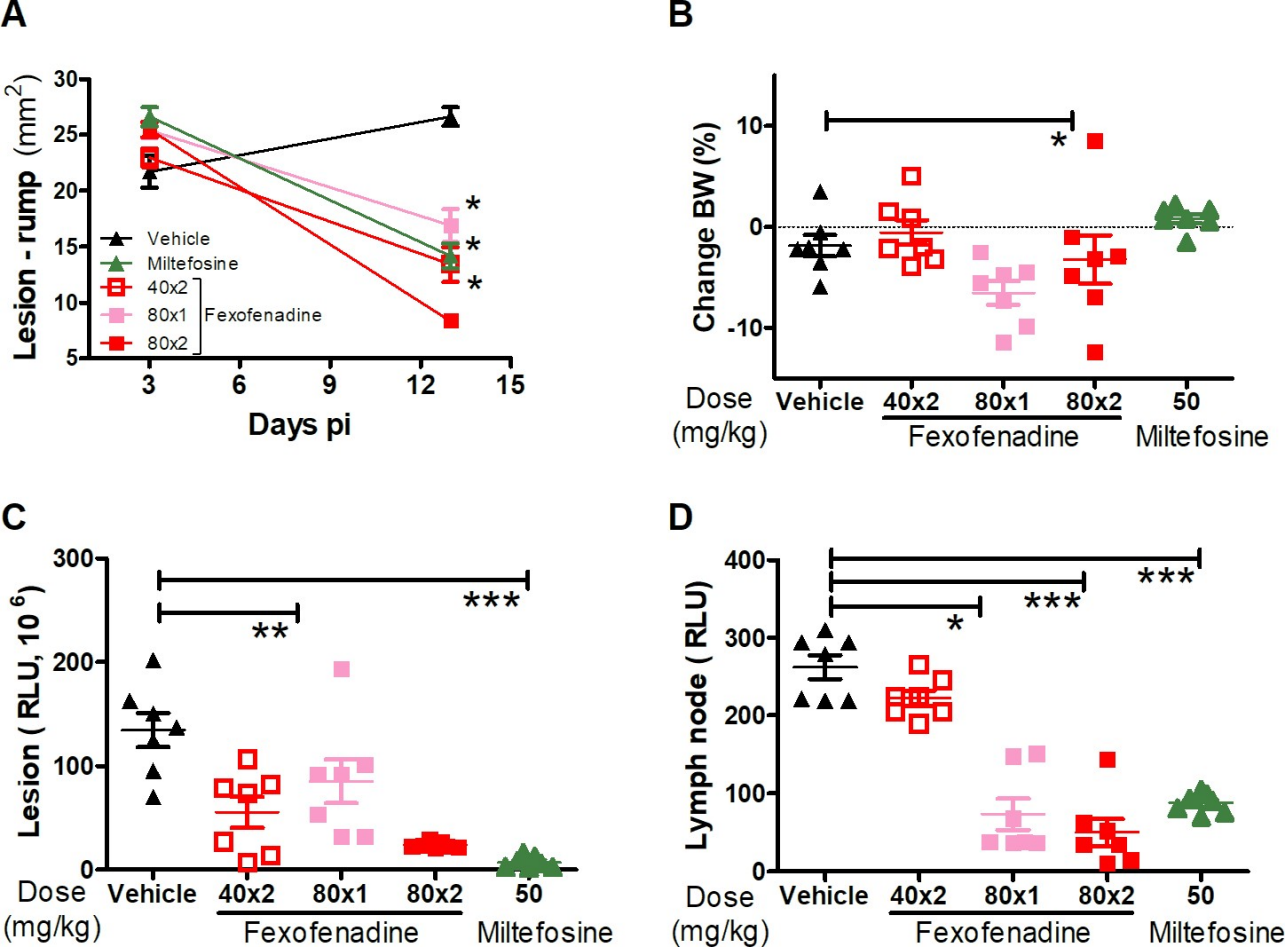
Evaluation of fexofenadine efficacy in BALB/c mice infected with *L*. *major*. Mice (n  =  7) were infected ID with 10^7^ metacyclic promastigotes of *L*. *major* transfected with the luciferase gene. Lesion size (area in mm2 = length x width) was measured using a digital caliper (A). Body weight change was used as a major sign of toxicity (B). The parasite load at the lesion site (C) and lymph nodes (D) was determined *in vivo* using the IVIS spectrum. Animals were treated PO with fexofenadine or vehicle (control) from day 3 to 10 p.i. The graphs show mean values and their standard deviation (SD). Representative data of 3 experiments. P values: *< 0.05; **< 0.01; ***< 0.001. The statistical significance of the data was determined using the t test.

**Fig 7:**
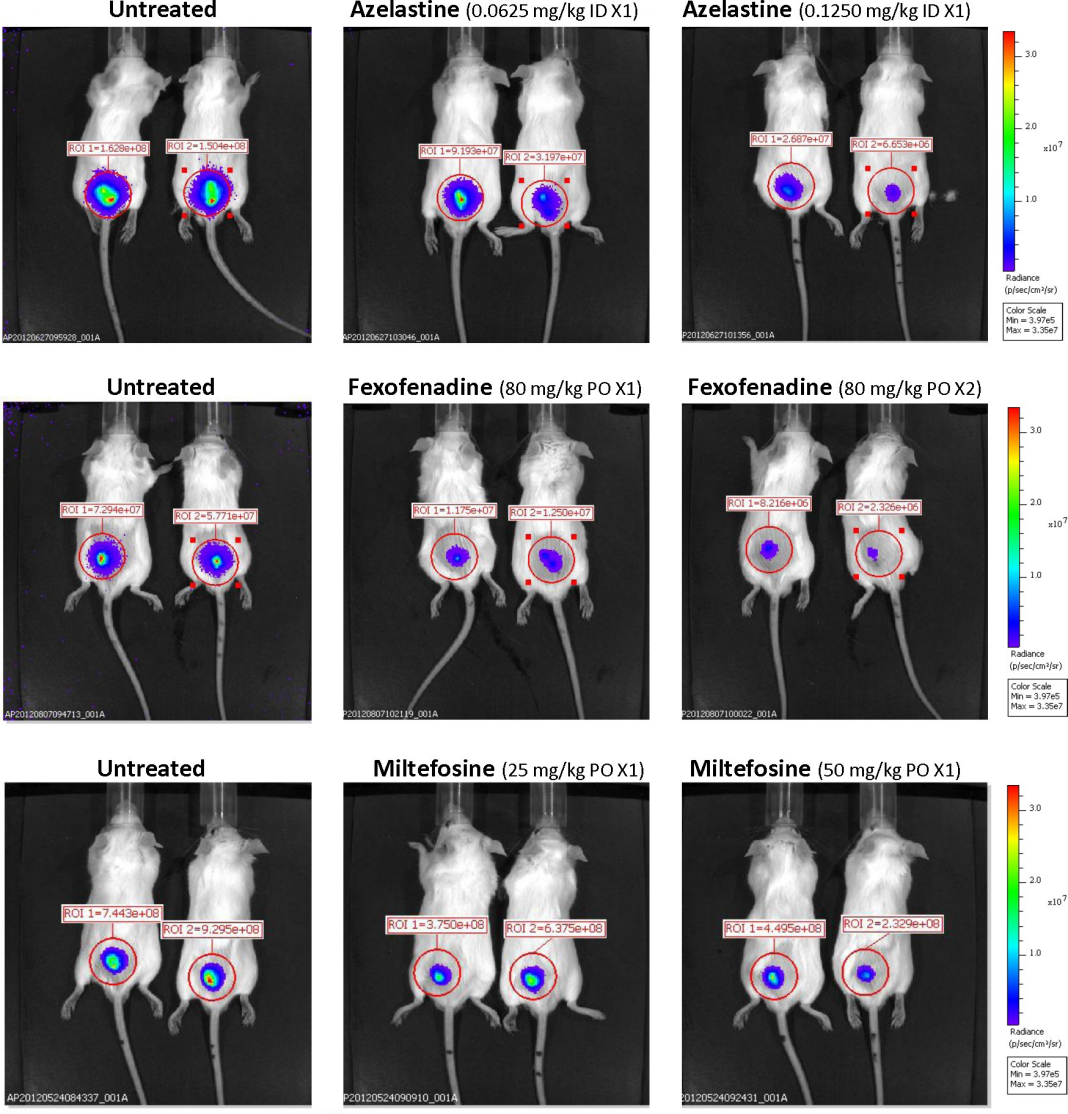
Representative IVIS images of mice treated for 10 days with different schedules and doses of azelastine or fexofenadine and the anti-leishmanial drug miltefosine.

Treatment of mice with FX at a dose of 80 mg/kg PO twice a day resulted in a 69% reduction in lesion size compared with the untreated control mice (**Fig 6A**). There was minimal loss of body weight (≤5%) and no other clinical side effect was found (**Fig 6B**). In addition, there was a significant reduction in parasite burden at the lesion site (82%) and draining lymph nodes (81%) as compared with the untreated control mice (p<0.0001 for both) (**Fig 6C and 6D**). The lower-dose regimens of FX demonstrated less pronounced anti-leishmanial efficacy. Mice treated twice a day PO with 40 mg/kg or once a day with 80 mg/kg showed a reduction in lesion size of 50% and 37%, respectively, compared with control mice (**Fig 6A and 6B**). These dosages also promoted reductions of parasite loads of up to 59% in lesions and 72% in lymph nodes in comparison with the control group (**Fig 6C and 6D**). Representative IVIS images of treatment outcomes are shown in **Fig 7**.

## Discussion

The present study used an *ex vivo* phenotypic assessment (killing of intracellular amastigotes) and *in vivo* therapeutic approach to determine the activity of antihistamine compounds against *L*. *major*. Our evaluations established that AZ and FX, 2^nd^ and 3^rd^ generation H1R antagonists, respectively, have significant anti-leishmanial activity compared with other H1R antagonists assessed in this study. The therapeutic potential was determined using the *in-vitro*-therapeutic-index (IVTI), which in addition to the compound’s anti-leishmanial activity, considers its cellular toxicity. For this reason, the IVTI from other H1R antagonists such as chloropyramine, cyproheptadine and mequitazine, which showed good EC_50_ were ranked as having lower therapeutic potential. Nevertheless, these compounds could still be considered as potential drug candidates since currently used compounds such as miltefosine and amphotericin B showed similarly low IVTIs. Furthermore, these compounds may provide clues to consider for lead optimization.

Other authors evaluated the activity of various H1RAs against *L*. *infantum* [19]. Different to our study, the *in vitro* system used infected spleen macrophages from hamsters and NCTC cells for determining IC_50_ and CC_50_, respectively. In that study, FX required >100 μM concentrations to achieve the IC_50_ value. The contrasting result of FX’s anti-leishmanial efficacy compared with our work could be due to the utilization of a different *ex vivo* system that includes multiple immune cell populations, or a different species of *Leishmania*. The anti-leishmanial activity in our *ex vivo* culture involves the cross talk of infected macrophages and T cells from lymph nodes, while the evaluations made by de Melo Mendes et al. [19] were performed using isolated splenic macrophages with no influence from lymphocytes and their cytokine production.

The high IVTI score of FX indicated that it was a good candidate drug for *in vivo* evaluation in the BALB/c-*L*. *major* model. Oral administration of the high dose (80 mg/kg) of FX given twice a day for 10 days showed significant reduction of lesion size and parasite numbers in the lesion and lymph nodes, with no observable side effects. On the other hand, the high single daily dose failed to reach significance, except for the reduction in parasite load in the lymph node. Pharmacokinetic studies in humans indicated that FX has a half-life (t_1/2_) of approximately 6 hours [33] stressing the importance of administering the drug twice a day, even at the high doses used in our study. It is conceivable that a slow release formulation given once a day could achieve an efficacy comparable to FX given twice daily.

The highly favorable IVTI of AZ prompted us to perform *in vivo* evaluations. We selected AZ for lesion treatment based on its local use as a nasal spray, while FX was given systemically (orally), which is the common route utilized in humans. The preclinical trial using BALB/c mice infected with *L*. *major* showed that three intralesional injections of AZ (0.0625 mg/mouse) delivered at 48 h intervals resulted in a non-significant decrease in lesion size or parasite burden as compared with untreated controls. However, both results reached statistical significance when the higher dose (0.125 mg/mouse) was used. The study suggested that AZ has anti-leishmanial activity as a local therapy, an approach utilized with some of the current anti-leishmanial compounds [34–36]. It is conceivable that intralesional treatments combining AZ with different local anti-leishmanial drugs could rapidly reduce inflammation and improve treatment schedules.

A potential limitation of this study is the lack of data on systemic AZ. It would be relevant for further studies to evaluate the systemic efficacy of AZ for cases in which multiple lesions are present or when there is increased risk of mucosal metastasis (e.g. *Leishmania Viannia* spp.). For this purpose, oral tablets instead of the commonly used intranasal spray would be the best therapeutic option. This alternative is supported by the lack of side effects observed in a multicentric clinical trial in which daily oral administration of 4 mg AZ for 21 days was used to treat chronic idiopathic urticaria [37]. Consequently, based on the good safety results of AZ, a similar high-dose regimen in combination with an anti-leishmanial drug could be assessed in preclinical studies for cutaneous leishmaniasis.

It is important to emphasize that the selection of H1R antagonists as anti-leishmanial drugs requires thorough *in vitro* determinations before a true lead could be identified. Pinto et al. [2014] evaluated several H1R antagonists against *L*. *infantum* but most of them showed efficacy only against the promastigote form at relatively high drug concentrations (15–84 μM), while the activity against intracellular amastigotes in hamster peritoneal macrophages was poor [38]. Furthermore, the CC_50_ using NCTC cells suggested that the cytotoxicity was an additional drawback. In that study, the anti- histamine cinnarizine yielded high killing capacity of *L*. *infantum* in mouse peritoneal macrophages, but in another study the compound failed to significantly decrease the parasite load in hamster spleen, the principal target organ [19, 38].

Our efficacy evaluations were performed using the *ex vivo* lymph node explant model [21], where drugs exert direct or indirect activity against amastigote-laden macrophages within the context of the immunologic milieu. The mechanism of action may include parasite targets or host signaling pathways. We found that AZ significantly decreased the expression of *H1R* and *IL-1ẞ*, suggesting that inhibition of this cell receptor modulates the production of a pro-inflammatory cytokine that may be disease-promoting [39, 40]. In fact, the suppressive effects of proinflammatory cytokines (TNF-α, IL-1-β, GM-CSF and IL-6) upon AZ administration has been described in *in vitro* experiments, animal models and humans [41, 42]. These results are supported by other studies showing that excessive activation of *H1R* (and *H4R*) result in a dysregulated inflammatory Th1 response and pro-inflammatory gene expression [43, 44]. Our observations are contrasting with those of Lima-Junior et al. [45] in which the inflammasome-driven *IL-1ẞ* production led to NOS2 production and resistance against *L*. *amazonensis* in C57BL/6 mice. This discrepancy could be partially explained by the negligible importance that the inflammasone had in *L*. *major* infection compared with other *Leishmania* species, as determined by the authors of the same study [45].

*In vitro* studies using enzymes (COX-1 and COX-2) or PBMCs from allergic patients showed that FX had a significant anti-inflammatory effect through inhibition of inflammatory mediators such as COX-1, COX-2, NF-kB-p50, CCR1, CCL5/RANTES and IL-1ẞ [46, 47]. Therefore, our results suggest that the anti-leishmanial activity of FX and AZ observed in the EVC was likely due to the anti-inflammatory activity mediated through H1R inhibition. Maximal anti-leishmanial activity occurred in the macrophage-T cell co-culture when the T cells were derived from the draining LN of a Leishmania-infected mouse, suggesting that the H1R inhibitor was promoting a more effective T cell response. There may be more than one pathway of anti-leishmanial activity of AZ since the drug had some suppressing effect against intracellular amastigotes in absence of lymphocytes. The drug was shown to alter mitochondrial function of *L*. *infantum* [19] and reversed antibiotic resistance by disrupting the membrane of Gram positive and Gram negative bacteria thereby facilitating cell penetration of bactericidal drugs [48–51]. A similar mechanism as chemo-sensitizer could be responsible for the successful therapy of patients infected with chloroquine- or amodiaquine-resistant *P*. *falciparum* when the H1R antagonist chlorpheniramine was added to treatment [16, 17].

Our study did not evaluate other histamine receptors (*H2R*, *H4R*) that could also be involved in *Leishmania* suppression. For example cimetidine, an *H2R* antagonist, was found to be effective for treating BALB/c mice infected with *Leishmania mexicana* when used alone or in combination with pentostam [52, 53]. Therefore, the additive or synergistic effect of using multiple histamine receptor antagonists still needs to be assessed.

Overall, our study suggested that AZ and FX should be further evaluated as viable alternatives to reduce toxicity and improve efficacy of cutaneous leishmaniasis treatment administered alone or in combination with current anti-leishmanial drugs.

### Disclaimer

Some of the data correspond to the PhD thesis of A. Peniche; Escuela de Ciencias Básicas, Facultad de Salud, Universidad del Valle (Cali, Colombia).

## Supporting information

S1 Fig Chemical structures of Histamine H1R antagonist compounds evaluated in this study. (TIF)
