# Supplementary figures and images for: Efficacy of histamine H1 receptor antagonists azelastine and fexofenadine against cutaneous Leishmania major infection
Source: PLoS Negl Trop Dis. 2020 Aug 10;14(8):e0008482. doi: 10.1371/journal.pntd.0008482 (PMC7449455; doi:10.1371/journal.pntd.0008482)

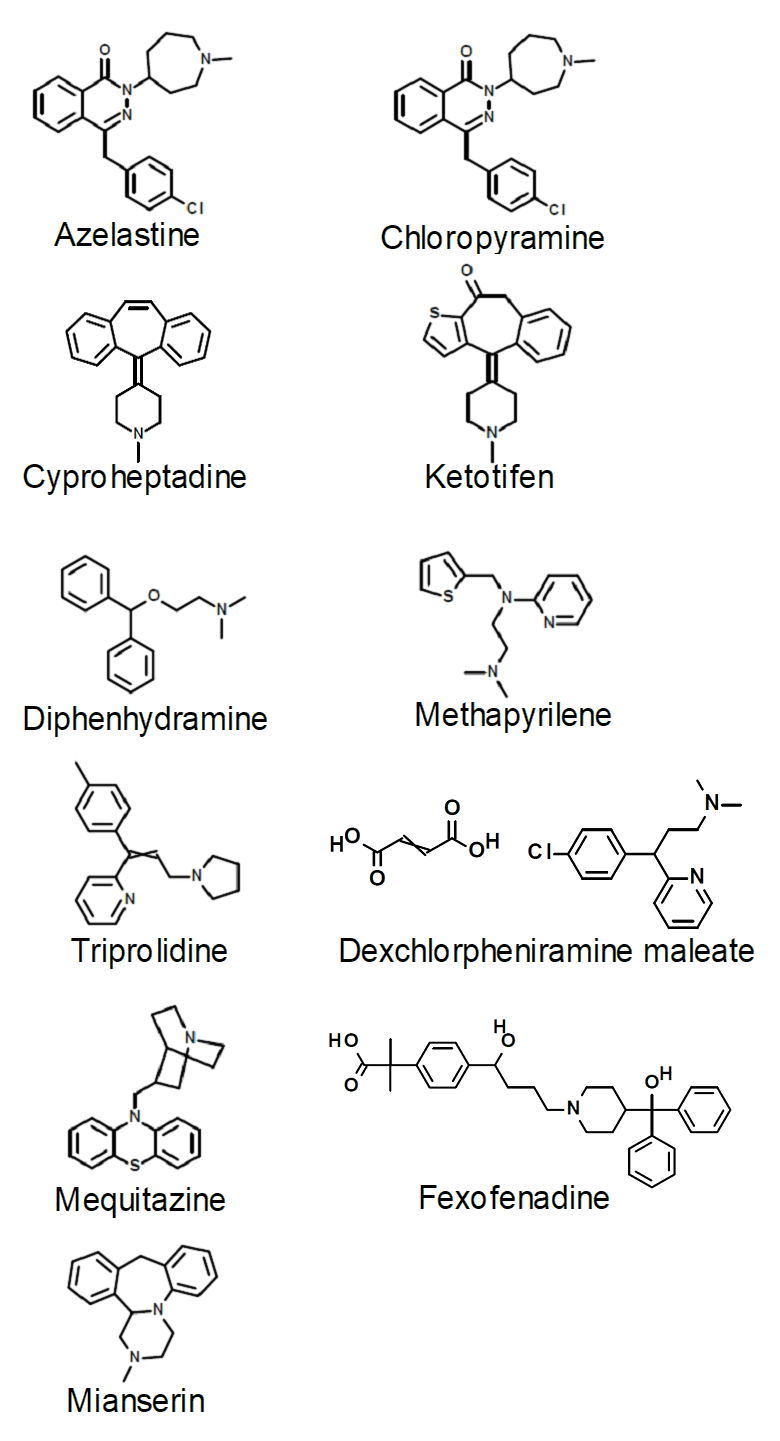

Supplement: S1 Fig — (TIF) [file pntd.0008482.s001.tif]
